# The Highly Selective 5-HT_2B_ Receptor Antagonist MW073 Mitigates Aggressive Behavior in an Alzheimer’s Disease Mouse Model

**DOI:** 10.3390/cells15030273

**Published:** 2026-02-01

**Authors:** Erica Acquarone, Saktimayee M. Roy, Agnieszka Staniszewski, Daniel Martin Watterson, Ottavio Arancio

**Affiliations:** 1 Taub Institute for Research on Alzheimer’s Disease and the Aging Brain, 630 West 168th Street, P&S 12-420D, New York, NY 10032, USA; ea2796@cumc.columbia.edu (E.A.); as2637@cumc.columbia.edu (A.S.); 2 Department of Pharmacology, Feinberg School of Medicine, Northwestern University, 320 E. Superior St., Chicago, IL 60611, USA; s-roy3@northwestern.edu (S.M.R.); d.m.watterson@gmail.com (D.M.W.); 3 Department of Pathology and Cell Biology, Columbia University, New York, NY 10032, USA; 4 Department of Medicine, Columbia University, New York, NY 10032, USA

**Keywords:** 5-hydroxytryptamine, 5-HT_2B_ receptor, serotonin, Alzheimer’s disease, neuropsychiatric syndrome, MW073

## Abstract

**Highlights:**

**What are the main findings?**

- MW073, a selective 5-HT_2B_ receptor antagonist, significantly reduces both the number and duration of aggressive attacks in male *Tg2576* mice.
- Treatment with MW073 effectively ameliorates aggressive behavior in male *Tg2576* mice.

**What are the implications of the main findings?**

- 5-HT_2B_ receptor antagonism may represent a targeted therapeutic strategy to reduce pathological aggression.
- Targeting 5-HT_2B_ receptors could provide a novel approach for managing aggression associated with Alzheimer’s disease–related pathology.

**Abstract:**

**Background:** Alzheimer’s disease (AD) is a multifactorial neurodegenerative disorder and the leading cause of dementia worldwide. Progressive synaptic dysfunction underlies declines in cognition, daily functioning, and the development of neuropsychiatric syndromes. Neuropsychiatric syndromes that include agitation and aggression affect 40–60% of patients and represent a major source of caregiver burden. Serotonin 5-HT_2B_ receptor levels are increased in the AD patient brain, and thus, treatment of AD animal models with the selective 5-HT_2B_ receptor antagonist MW073 in prevention or disease stage paradigms attenuates Aβ- or tau-induced dysfunction. **Methods:** We investigated the effects of MW073 treatment on the aggressive behavior of *Tg2576* mice in a resident–intruder assay. **Results:** MW073 treatment significantly reduced aggressive behavior in male *Tg2576* mice. **Conclusions:** MW073 efficacy in treating aggression in *Tg2576* mice implicates 5-HT_2B_ receptor-mediated signaling in AD neuropsychiatric symptoms as well as cognitive and behavioral dysfunction.

## 1. Introduction

Dementia, with Alzheimer’s disease (AD) and related diseases (ADRD) as its predominant form, is an increasing global health challenge. Cognitive impairment and neuropsychiatric syndromes (NPSs) are the clinical hallmarks that generate a collective impact on patients, families, and caregivers, as well as causing major public health and economic effects. NPSs affect patients’ quality of life across their life spans and are a clinical concern due to their impacts on cognition, social interactions, and non-pharmacological interventions. NPSs such as anxiety and agitation are clinical presentations common to diverse neurodegenerative diseases, neurodevelopment complications, and brain injury sequelae [1,2]. Agitation associated with dementia is characterized by excessive motor activity and aggression [3,4]. However, there is a lack of safe, effective dual-action therapies for agitation and cognition. Due to the lack of approved therapies, a variety of drugs such as anticonvulsants, antipsychotics, selective serotonin reuptake inhibitors (SSRIs), atypical antidepressants, sedatives, anti-dementia drugs, dextromethorphan, and cannabinoids are used to manage neuropsychiatric symptoms such as agitation, despite lacking well-defined dosing, safety, and efficacy information [5]. Two approved drugs for agitation that are widely used, but which have safety risks that require close monitoring, are brexpiprazole and risperidone, multi-target drugs acting on the serotoninergic and dopaminergic neurotransmitter systems implicated in AD-associated agitation [6,7]. Consistent with the proposed monoamine neurotransmitter mechanism in AD-related NPS, there is a loss of serotoninergic (5-hydroxytryptamine; 5-HT) neurons in the raphe nucleus and the appearance of neurofibrillary tangles in a region rich in 5-HT neurons, the dorsal raphe nucleus [8,9,10,11]. Further, 5-HT_2B_ receptor (5-HT_2B_R) levels are increased in AD patient brains [12], like they are in stroke and amyotrophic lateral sclerosis [13,14], and human *HTR2B* gene variants are implicated in psychiatric syndromes [15]. Clearly, there is a need to develop more selective, safer, and more effective candidates for at-risk dementia patient populations across multiple diseases, and the serotoninergic 5-HT_2B_R-regulated pathways are rational targets to explore.

We directly addressed this challenge with the development of MW073, a unique first-in-class selective inhibitor of serotonin 5-HT_2B_ receptor (5-HT_2B_R) activation and arrestin recruitment [16]. MW073 is an orally bioavailable candidate with a promising safety profile and low drug–drug interaction risk that attenuates amyloid- and tau-induced synaptic and behavioral dysfunction when used in a preventive or disease stage treatment paradigm. MW073 avoids key off-target liabilities, particularly 5-HT_2B_R agonist activity, that have contributed to withdrawal or black-box warnings in some approved drugs. MW073 inhibits 5-HT_2B_R signaling via the Gq-PLCβ2 pathway and β-arrestin recruitment in both human and mouse receptor systems, a mechanism associated with downstream biomarkers in neurodegenerative and neuropsychiatric disorders. MW073 provides a novel precision pharmacological tool with future potential for treating AD and neuropsychiatric syndromes.

MW073’s selective modulation of serotonergic signaling preserves synaptic function and restores behavioral outcomes in multiple disease models. To probe the potential extension of MW073 to neuropsychiatric syndromes, we evaluated MW073 treatment of aggressivity in an established AD-relevant animal model, the *Tg2576* mouse. Our objective was to test the hypothesis that MW073 regulation of aggressive behavior could be a potential therapeutic approach to managing AD-associated NPSs.

## 2. Materials and Methods

### 2.1. Animals and Housing Conditions

The study utilized *Tg2576* mice and nTg littermates. Intruder animals were *C57Bl/6*. *Tg2576* mice overexpress human mutant *APP* (isoform 695) containing the double mutation *K670N, M671L* (Swedish mutation) under the control of the hamster prion protein promoter. They are characterized by elevated levels of Aβ and ultimately amyloid plaques. They were obtained from a mouse colony that was established thanks to the generous gift of Karen Hsiao-Ashe. The animals were maintained on a 12 h light/12 h dark cycle, in a temperature- and humidity-controlled room. Food and water were available ad libitum. Mice were allocated to a specific treatment and paradigm by a randomization procedure. Investigators who performed the experiments were blind with respect to genotype and treatment.

### 2.2. Treatment Solution

The MW073 compound for treatment in behavioral testing was diluted in 10% Propylene Glycol (Sigma-Aldrich, St. Louis, MI, USA, P4347) MilliQ quality water, and 0.1% formic acid to prepare a stock solution. At the time of the experiment, the compound was diluted in sterile saline. MW073 was administered once daily by intraperitoneal injection (5 mg/kg) for 21 consecutive days prior to behavioral testing.

### 2.3. Experimental Design

**Figure 1:**
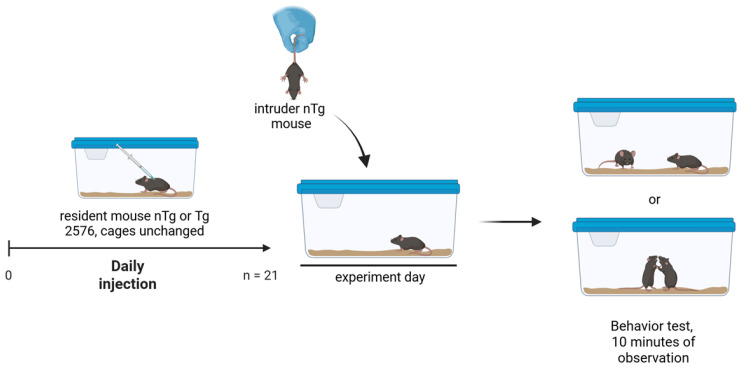
Experimental design. Resident mice (*nTg* or *Tg*) were in isolated cages during the 21 days of injections. On the test day, an intruder *nTg* mouse was added to the resident cage, and the social behavior of the animals was observed and recorded for 10 min directly after the introduction of the intruder mice. (Created in Biorender. Erica Acquarone (https://BioRender.com under Columbia license (2025)).

This study aimed to elucidate the effect of the 5HT_2B_ receptor antagonist MW073 on mouse aggressivity. Mice’s aggressive behavior was assessed by means of a combined isolation-induced and resident–intruder paradigm. For this purpose, mice were isolated for 3 weeks. The mice were single-housed in their standard cages and left undisturbed during the entire isolation period. Meanwhile, no fresh bedding material was provided to ensure that the area would become their own territory and to evoke aggressive behavior upon intrusion by another mouse of the same sex. After 3 weeks of isolation, mice were allowed to adapt to the observation room in their home cage for at least 1 h prior to testing. A group-housed male *C57Bl/6* mouse was introduced into the resident’s home cage. Only the behavior of resident mice was analyzed. The second mouse was classified as an intruder. To distinguish the intruder from the resident mouse, the intruder was marked with a black sign on the tail. The behavior was recorded for 10 min (Figure 1).

### 2.4. Observation of Social Behavior

During a 10 min observation period, the observer, who was blind to the mouse’s genotype, evaluated animal aggressivity. As an index of social behavior and aggressivity, we used mainly pouncing/chasing behaviors (chasing, attacking, and escalated fighting) from the resident mice, discriminating against approaches by the intruder, as well as facial/body sniffing, and ano-genital sniffing from resident mice. The observer recognized defensive behavior such as avoiding, fleeing, and defensive upright posture [17]. The number/severity of physical encounters were closely monitored, and the mice were separated if any encounter was severe enough to potentially cause injury. We quantified the number of attacks, the total duration of aggressive episodes, the duration of the first attack, and the latency to the first attack, defined as the time elapsed from the introduction of the intruder mouse into the resident’s cage to the occurrence of the first aggressive attack. The number of encounters and latency encounters were scored using a stopwatch, and every test was recorded (Noldus Information Technology, Wageningen, The Netherlands). Also, during analysis, the observer was blind to the mice’s genotype treatment.

### 2.5. Open Field Control Experiment

Spontaneous locomotor activity and anxiety-like behavior were assessed using the Open Field test. Mice were individually placed in the center of a square arena (40 cm × 40 cm, walls 40 cm high) made of white Plexiglas. The test was conducted in a quiet room to minimize external stressors. The animals were allowed to freely explore the arena for 10 min. Behavioral activity was recorded using a top-mounted video camera and analyzed with Activity Motor by MedAssociates Inc. (St. Albans, VT, USA, SOF-812 DOC.101 Rev. 1.3). The arena was virtually divided into two zones: a central zone (20 × 20 cm^2^) and a peripheral zone. The parameters analyzed included total distance traveled (cm), mean velocity (cm/s), time spent in the center (s), number of entries into the center, rearing behavior, and immobility time. The arena was thoroughly cleaned with 70% ethanol between sessions to eliminate olfactory cues. To assess habituation, mice were exposed to the same arena for two consecutive days under identical conditions. Changes in locomotor activity and center-related parameters between Day 1 and Day 2 were used as indicators of habituation and exploratory memory. Animals were acclimated to the testing room for at least 30 min prior to the session. Behavioral scoring was conducted blind to treatment conditions.

### 2.6. Statistical Analysis

Investigators who performed the experiments were blinded with respect to treatment and genotype. The data were scored as the total duration of each behavior performed during the 10 min observation period. The comparisons between *Tg* males and females, *Tg* and *nTg* mice, as well as *Tg* mice treated with vehicle and *Tg* treated with MW073 as a single variable, were carried out using Student’s *t*-test (2-tailed) and one-way ANOVA by GraphPad Prism10.

## 3. Results

### 3.1. Tg2576 Males Are More Aggressive than Tg Females

**Figure 2:**
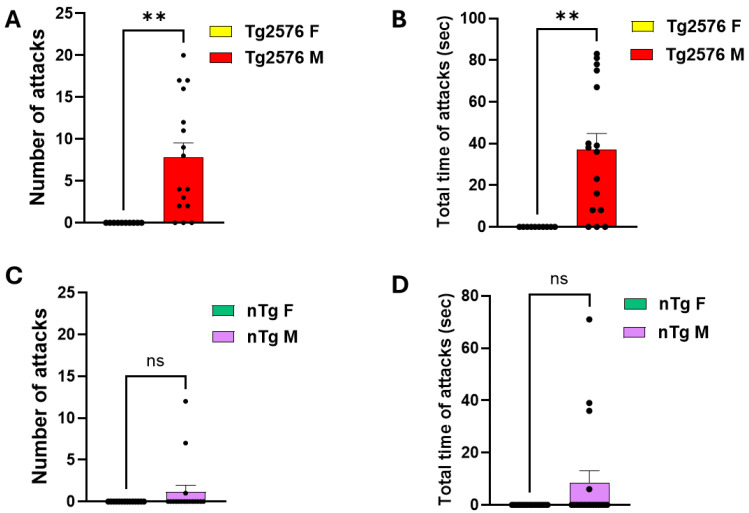
*Tg2576* males are more aggressive than *Tg2576* females. (**A**,**B**) Male *Tg2576* mice displayed a markedly greater number of attacks (**A**) and total attack duration (**B**) compared to female *Tg2576* mice. Specifically, *Tg2576* females showed no attacks (0), whereas *Tg2576* males averaged 7.81 ± 1.73 attacks (*p* = 0.0016); and total attack time was 0 sec in females versus 37 ± 7.77 s in males (*p* = 0.0010). These experiments were performed on 10 *Tg2576* female mice and 16 *Tg2576* male mice. The average age of females was 262.3 ± 29.21 days, whereas the average age of males was 294.56 ± 34.55 days (*p* = 0.3482). (**C**,**D**) Male *nTg* mice displayed an increased number of attacks (**C**) and longer total attack duration (**D**) compared with *nTg* females; however, these differences did not reach statistical significance. Specifically, *nTg* females showed no attacks (0), whereas *nTg* males averaged 1.42 ± 1.07 attacks (*p* = 0.1892); and total attack time was 0 sec in females versus 8.28 ± 6.28 s in males (*p* = 0.1082). These experiments were performed on 14 *nTg* females and 17 *nTg* males. The average age of females was 241.08 ± 50.51 days, whereas the average age of males was 246.17 ± 45.83 days (*p* = 0.9421). Standard errors and results from individual experiments are shown within the column bars in this and the following figures. (** *p* < 0.01, ns = not significant).

Our first objective was to evaluate potential sex differences in aggressive behavior in *Tg2576* mice. In the resident–intruder paradigm, no *Tg2576* resident females (0/16) initiated attacks against intruder females during the 10 min testing period. In contrast, 13 out of 16 *Tg2576* resident males exhibited overt aggression toward intruder males (Figure 2A,B). Quantitative analysis revealed that both the frequency of attacks and the cumulative attack duration were significantly higher in Tg2576 males compared to females (*p* < 0.001; Figure 2A,B). Similarly, non-transgenic (*nTg*) females showed no attacks compared to *nTg* males (Figure 2C,D). Given the pronounced male-specific aggressive phenotype, all subsequent pharmacological experiments with MW073 were conducted in *Tg2576* male residents.

### 3.2. Aggressive Behavior Test in Tg2576 and Non-Transgenic (nTg) Male Littermates

**Figure 3:**
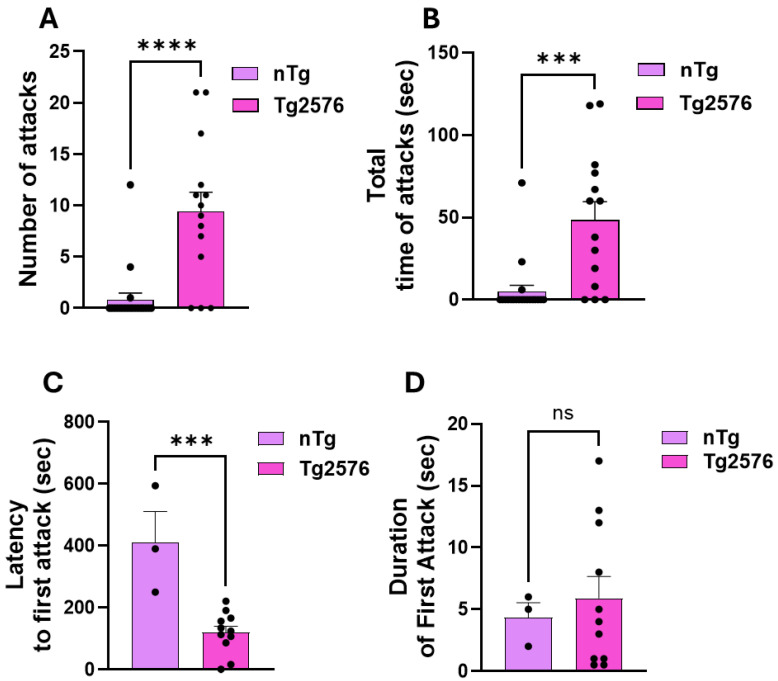
*Tg2576* resident males are more aggressive than nTg resident males. (**A**,**B**) Both the mean number of attacks (**A**) and the mean total attack duration (**B**) were dramatically higher in *Tg2576* animals compared to non-transgenic (*nTg*) mice (number of attacks: *nTg* = 0.85 ± 0.62, *Tg* = 9.43 ± 1.86, *p* < 0.0001; total attack time: *nTg* = 5.00 ± 9.46 s, *Tg* = 48.43 ± 11.08 s, *p* = 0.0002). (**C**) *nTg* resident males exhibiting an attack displayed longer latency to the first attack compared to Tg2576 mice *(nTg* = 411.33 ± 99.87 s, *Tg* = 119.54 ± 20.27 s, *t*-test *p* = 0.0004). (**D**) *nTg* residents exhibiting an attack showed slightly shorter, though not statistically significantly, first-attack duration compared to Tg2576 residents (*nTg* = 4.33 ± 1.20 s, Tg = 5.91 ± 1.74 s, *p* = 0.6590). These experiments were performed on 20 *nTg* mice and 14 *Tg2576* male mice. Analyses of attack initiation parameters (latency and first-attack duration) for this figure and the following one were limited to animals that exhibited at least one aggressive attack. The average age of *nTg* mice was 224.2 ± 40.22, whereas the average age of the *Tg2576* was 181 ± 13.28 (*p* = 0.3897). (*** *p* < 0.001, **** *p* < 0.0001, ns = not significant).

Our next goal was to compare aggressiveness in *Tg2576* mice compared to *nTg* males. We found that only 3 out of 20 *nTg* resident males exhibited aggression toward intruder males during the 10 min resident–intruder test, whereas 11 out of 14 *Tg2576* residents initiated attacks (Figure 3A–D). Both the average number of attacks and attack duration were higher in Tg2576 residents than *nTg* littermates (*p* < 0.001; Figure 3A,B). For parameters related to attack initiation, including latency to the first attack and duration of the first attack, analyses were restricted to animals that displayed an aggressive attack during the testing period. Among the animals that exhibited an attack, the latency to the first attack was markedly reduced in *Tg2576* mice, which began aggression at approximately 2 min following intruder introduction, compared to ~7 min in *nTg* controls (*p* < 0.01; Figure 3C). In the same animals, the duration of the initial attack was slightly longer in *Tg2576* mice relative to *nTg*, but this difference did not reach statistical significance (Figure 3D).

### 3.3. Aggressive Behavior Is Reduced in Male Tg2576 Mice Treated with MW073 Compared to Tg2576 Mice Treated with Vehicle

**Figure 4:**
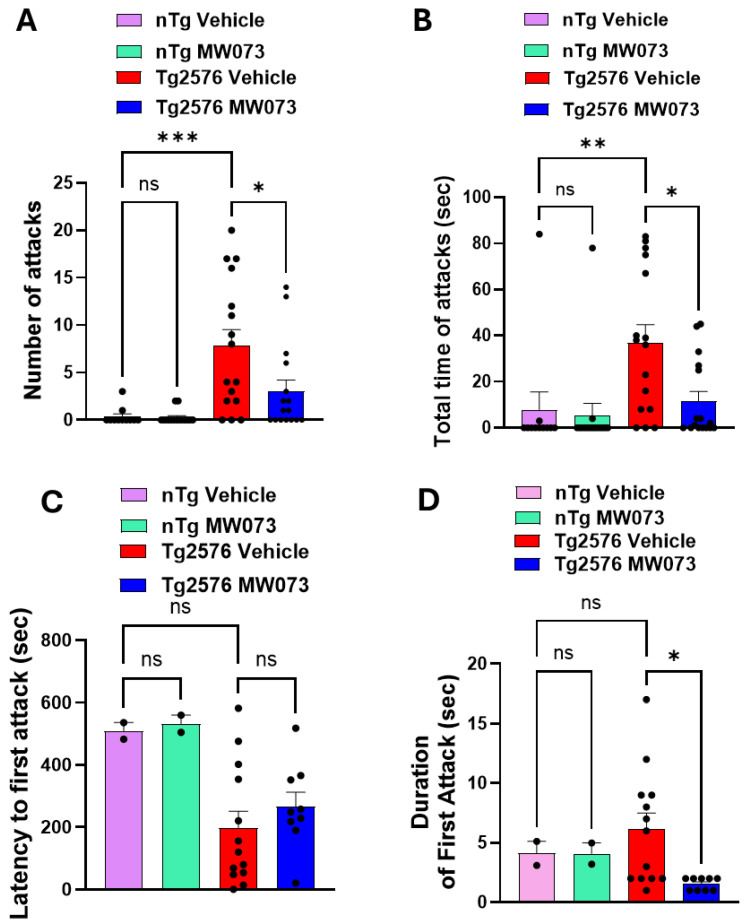
*Tg2576* mice treated with MW073 showed amelioration of aggressive behavior. (**A**,**B**) The number of attacks (**A**) and the total attack time (**B**) of *Tg2576* resident males treated with MW073 were reduced compared to *Tg2576* treated with vehicle (number of attacks: MW073 = 3.06 ± 1.15, vehicle = 7.81 ± 1.72; *p* = 0.029; total attack time: MW073 = 11.56 ± 4.23, vehicle = 37.00 ± 7.77; *p* = 0.0074). In interleaved experiments, *nTg* males treated with MW073 exhibited the same number of attacks as vehicle-treated *nTg* males. The total attack time was similar between the two groups on *nTgs* (number of attacks: MW073 = 0.28 ± 0.18, vehicle = 0.36 ± 0.28; *p* = 0.815; total attack time: MW073 = 5.86 ± 5.37, vehicle = 7.90 ± 7.61; *p* = 0.825). Number of attacks: 1-way ANOVA followed by Bonferroni’s comparisons F(3,54) = 9.559 *p* < 0.0001; *Tg2576* vehicle vs. *Tg2576* MW073 *p* = 0.011; *nTg* vehicle vs. *nTg* MW073 *p* > 0.999; *Tg2576* Vehicle vs. *nTg* vehicle *p* = 0.0002). Total attack time: 1-way ANOVA followed by Bonferroni’s comparisons F(3,54) = 5.689 *p* = 0.0018; *Tg2576* vehicle vs. *Tg2576* MW073 *p* = 0.012; *nTg* vehicle vs. *nTg* MW073 *p* > 0.999; *Tg2576* vehicle vs. nTg vehicle *p* = 0.009) (**C**) *Tg2576* resident males treated with MW073 displayed longer latency tendency to the first attack compared to *Tg2576* treated with vehicle (MW073 = 266.8 ± 34.32 s, vehicle = 198.2 ± 48.17 s; *p* = 0.3708). *nTg* males treated with MW073 displayed comparable latency to the first attack as vehicle-treated *nTg* males (MW073 = 532.50 ± 10.04 s, vehicle = 509.50 ± 11.30 s; *p* = 0.608. One-way ANOVA followed by Bonferroni’s comparisons F(3,54) = 0.8137 *p* = 0.4919; *Tg2576* vehicle vs. *Tg2576* MW073 *p* > 0.999; *nTg* vehicle vs. *nTg* MW073 *p* > 0.999 *Tg2576* vehicle vs. nTg vehicle *p* > 0.999). (**D**) *Tg2576* resident males treated with MW073 showed shorter duration of the first attack compared to *Tg2576* treated with vehicle (MW073 = 1.556 ± 0.17, vehicle = 6.154 ± 1.33 s; *p* = 0.01). *nTg* males treated with MW073 displayed a comparable duration of the first attack to vehicle-treated *nTg* males (MW073 = 4.10 ± 0.32 s, vehicle = 4.16 ± 0.43 s; *p* = 0.992). These experiments were performed on 16 *Tg2576* mice, either treated with vehicle or MW073, 11 *nTgs* treated with vehicle, and 15 *nTgs* treated with MW073. One-way ANOVA followed by Bonferroni’s comparisons F(3,54) = 8.655 *p* < 0.0001; *Tg2576* vehicle vs. *Tg2576* MW073 *p* = 0.001; *nTg* vehicle vs. *nTg* MW073 *p* > 0.999, *Tg2576* vehicle vs. nTg vehicle *p* = 0.001) The average age of *Tg2576* mice treated with vehicle was 294.56 ± 34.550, whereas the average age of *Tg* mice treated with MW073 was 280.81 ± 32.954. The average age of *nTg* mice treated with vehicles was 261.10 ± 32.19, whereas the average age of *nTg* mice treated with MW073 was 253.00 ± 34.33. One-way ANOVA followed by Bonferroni’s comparisons F(3,54) = 0.2933 *p* = 0.8296; *Tg2576* vehicle vs. *Tg2576* MW073 *p* > 0.999; *nTg* vehicle vs. *nTg* MW073 *p* > 0.999, *Tg2576* vehicle vs. *nTg* vehicle *p* > 0.999). (* *p* < 0.05, ** *p* < 0.01, *** *p* < 0.001, ns = not significant).

Next, we determined whether the 5HT_2B_ receptor antagonist MW073 was capable of reducing the aggressive behavior of *Tg2576* male mice. Animals were treated with the antagonist for 3 weeks (daily, i.p., 5 mg/kg) prior to performing the aggressivity test. Our data revealed that *Tg2576* resident males treated with the serotonin antagonist (*n* = 16) showed less aggressivity than *Tg2576* mice treated with vehicle (*n* = 16) during the 10 min resident–intruder test sessions (Figure 4A–D). Specifically, the number of attacks and the duration of total attacks decreased in *Tg2576* treated with MW073 compared to *Tg* mice treated with vehicle (Figure 4A,B). Moreover, among the animals that exhibited an attack, resident *Tg2576* mice treated with vehicle started the first attack after ~3 min when the intruder was inserted into the cage. By contrast, resident *Tg2576* mice treated with MW073 started the first attack after ~4.45 min (Figure 4C). Most importantly, the duration of the first attack in *Tg* mice treated with MW073 was reduced (Figure 4D).

### 3.4. No Significant Differences Were Observed in the Open Field Test Among Male nTg Mice Treated with Vehicle or MW073 and Male Tg Mice Treated with Vehicle or MW073

**Figure 5:**
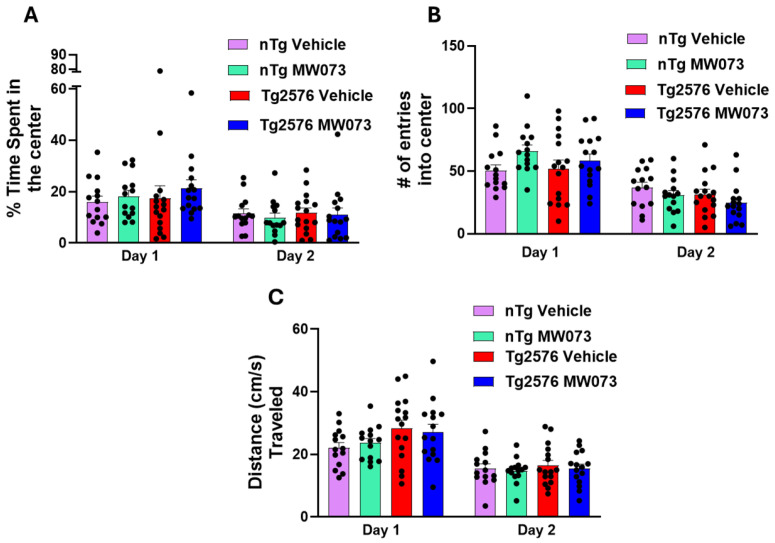
*Tg2576* and *nTg* mice treated with MW073 or vehicle did not show any difference in the open field test. (**A**) *Tg2576* and *nTg* males treated with MW073 showed no differences compared to *Tg2576* and *nTg* treated with vehicle during the test (day 1: *nTg* vehicle = 16.34 ± 2.37 s; *nTg* MW073 = 18.2 ± 2.27 s, *Tg2576* vehicle = 17.48 ± 4.75 s; *Tg2576* MW073 = 21.41 ± 3.15 s; *p* = 0.7149. Day 2: *nTg* vehicle = 11.52 ± 1.74 s; *nTg* MW073 = 9.83 ± 1.80 s, *Tg2576* vehicle = 11.71 ± 1.92 s; *Tg2576* MW073 = 10.96 ± 2.69 s; *p* = 0.7149). (**B**) *Tg2576* and *nTg* males treated with MW073 showed no differences compared to *Tg2576* and *nTg* treated with vehicle during the test (day 1: *nTg* vehicle = 51.62 ± 4.52; *nTg* MW073 = 66.07 ± 4.84, *Tg2576* vehicle = 52.12 ± 6.80; *Tg2576* MW073 = 58.34 ± 5.35; *p* = 0.2093. Day 2: *nTg* vehicle = 36.92 ± 4.21; *nTg* MW073 = 30.78 ± 3.72, *Tg2576* vehicle = 31.37 ± 4.15; *Tg2576* MW073 = 24.67 ± 4.02; *p* = 0.2217) (**C**) *Tg2576* and *nTg* males treated with MW073 showed no differences compared to *Tg2576* and *nTg* treated with vehicle during the test (day 1: *nTg* vehicle = 22.07 ± 1.66 cm/s; *nTg* MW073 = 23.63 ± 1.45 cm/s, *Tg2576* vehicle = 28.97 ± 2.92 cm/s; *Tg2576* MW073 = 27.72 ± 2.63 cm/s; *p* = 0.1970. Day 2: *nTg* vehicle = 15.48 ± 1.51 cm/s; *nTg* MW073 = 14.78 ± 1.07 cm/s, *Tg2576* vehicle = 17.41 ± 1.68 cm/s; *Tg2576* MW073 = 15.38 ± 1.51 cm/s; *p* = 0.8698.) These experiments were performed on 14 *nTg* mice either treated with vehicle or MW073, 15 *Tg2576* treated with vehicle, and 16 *Tg2576*. (# = number of center entries).

Changes in locomotor activity, exploratory behavior, or anxiety represent potential non-specific confounds that could affect the aggressive phenotype. To exclude the possibility that the beneficial effects of MW073 in *Tg2576* mice were secondary to changes in locomotor activity, anxiety-like behavior, or exploratory drive, we assessed animals in the open field test after 3 weeks of daily treatment with the 5-HT_2B_ receptor antagonist (5 mg/kg, i.p.). Analysis of time spent in the center versus the periphery of the arena revealed no significant differences between MW073- and vehicle-treated groups, either in *nTg* males (*n* = 14/group) or *Tg2576* males (MW073, *n* = 15; vehicle, *n* = 16) during the 10 min sessions on days 1 and 2 (Figure 5A). As expected, all groups spent less time in the center on day 2 relative to day 1, reflecting reduced anxiety and successful memory-based habituation.

Similarly, the number of center entries, another measure of anxiety-like behavior, did not differ between MW073- and vehicle-treated animals in either genotype (Figure 5B). Again, all groups exhibited reduced center entries on day 2, consistent with reduced anxiety and enhanced exploration drive.

Finally, total distance traveled, a measure of general locomotor activity, was comparable across treatment groups in both *nTg* and *Tg2576* males (Figure 5C). All groups showed decreased activity on day 2 relative to day 1, consistent with habituation to the test environment. Taken together, these data indicate that MW073 does not alter locomotor function, anxiety-like behavior, or exploration activity, supporting the conclusion that its effects on aggression in *Tg2576* mice are not confounded by changes in these parameters.

## 4. Discussion

There are two key aspects of this report. First, we demonstrate that male *Tg2576* mice exhibit a pronounced aggressive phenotype compared to both female *Tg2576* mice and male *nTg* littermates. Using the resident–intruder paradigm, *Tg2576* males showed a markedly higher frequency and duration of attacks and a shorter latency to initiate aggression, highlighting a sex- and genotype-specific increase in aggressive behavior. Second, we demonstrated that MW073 treatment significantly reduced the number and duration of attacks in *Tg2576* males without affecting latency to the first attack. These effects were not attributable to changes in locomotor activity, anxiety-like behavior, or exploratory drive, as assessed in the open field test, and are consistent with our cognitive findings, in which MW073 improved behavioral outcomes without producing nonspecific performance effects. Collectively, these findings identify *Tg2576* males as a robust preclinical screening model for aggression associated with AD-related pathology and extend the efficacy of MW073 in preventing or attenuating amyloid- and tau-induced synaptic and cognitive dysfunction to AD [16]-associated agitation, a key neuropsychiatric symptom common to several chronic diseases, as well as morbidities associated with acute brain injuries.

MW073 is a uniquely selective, mechanistically validated, and safety-derisked therapeutic candidate targeting 5-HT_2B_R that is a potential future precision medicine approach to treating NPSs with cognitive dysfunction [16]. MW073 represents a different therapeutic category. MW073: (1) targets a receptor upregulated in AD [12], amyotrophic lateral sclerosis (ALS) [13], and stroke-related NPS [14]; (2) directly reverses synaptic dysfunction caused by Aβ and tau oligomers [16]; (3) offers non-sedating, non-neuroleptic anti-aggression potential; (4) avoids metabolic, neurological, and cardiovascular liabilities of current agents; (5) provides a mechanistically rational intervention in diseases where 5-HT_2B_ elevation is pathophysiologically meaningful [16]. Taken in its entirety, MW073 offers a novel potential treatment of agitation, irritability, anxiety, or aggression in neurodegenerative disease populations.

Neuropsychiatric symptoms such as agitation, aggression, irritability, anxiety, and behavioral dysfunction are highly prevalent in AD, ALS, post-stroke conditions, traumatic brain injury, and other neurodegenerative disorders. They are associated with patient distress, caregiver burden, increased institutionalization rates, and elevated healthcare utilization. Despite their clinical importance, no FDA-approved therapies directly target the mechanistic drivers of these neuropsychiatric presentations in neurodegenerative diseases. Current management relies on diverse multi-target drugs that offer modest and inconsistent benefit and carry substantial safety risks, especially in older or severe neuropsychiatric populations. They include atypical antipsychotics, SSRIs, mood stabilizers, benzodiazepines, and beta-blockers. Atypical antipsychotics, such as risperidone, are frequently used for aggression or agitation. Their inhibitory targets include dopamine D2, 5-HT_2A_, H1, and α1 receptors. Key liabilities include extrapyramidal symptoms, sedation, metabolic syndrome, QT prolongation, and orthostatic hypotension. There are black box warnings for increased mortality in elderly dementia patients. SSRIs and serotonin and norepinephrine reuptake inhibitors (SNRIs) increase serotonin globally, affecting all 5-HT receptor subtypes with modest, slow-onset benefits for agitation and exhibit undesired side effects, including gastrointestinal problems, sexual dysfunction, and hyponatremia. Mood stabilizers such as valproate and carbamazepine generate broad CNS dampening through GABA enhancement and ion channel modulation with risks of hepatotoxicity, teratogenicity, sedation, and tremor. Overall, existing approved drugs manage symptoms vs. underlying synaptic or systems-level dysfunctions. In contrast to existing agents, MW073 provides targeted modulation of a receptor upregulated in AD, ALS, and post-stroke conditions.

Several serotonin receptor subtypes are expressed in distinct brain regions and contribute to the fine-tuning of complex emotional and social behaviors, including aggression. Consistent with our findings, a growing body of evidence indicates that the serotonergic system plays a central role in modulating aggression across various behavioral paradigms. Notably, different serotonin receptor subtypes have been associated with distinct aspects of aggressive behavior. For instance, 5-HT_1A_ and 5-HT_1B_ receptors located in the prefrontal cortex (PFC) modulate reactive aggression in the resident–intruder test [18,19,20]. Local microinjection of WAY-100635 (a 5-HT_1_A receptor antagonist and dopamine D4 agonist) and SB-224289 (a mixed serotonin receptor antagonist with selectivity for 5-HT1b) into the PFC significantly alters both the frequency and intensity of aggressive episodes, underscoring receptor- and region-specific serotonergic control of impulsive aggression. Similarly, members of the 5-HT_2_ receptor family (5-HT_2A_ and 5-HT_C_) have been implicated in aggression regulation, although their effects appear context-dependent: while 5-HT_2C_ receptor activation typically suppresses aggression, 5-HT_2A_ receptor activation may enhance it [21]. Unlike other 5-HT_2_ family members, the role of 5-HT_2B_ receptors in aggression has been minimally studied. Our findings now establish 5-HT_2B_ as an important and previously unappreciated regulator of aggressive behavior in AD.

Together, these results position 5-HT_2B_ receptor antagonism as a compelling mechanistic strategy for mitigating neuropsychiatric symptoms in AD. By demonstrating that MW073 selectively reduces pathological aggression in *Tg2576* males, without inducing sedation, dampening locomotion, or altering anxiety-like behavior, our study provides clear evidence that targeted serotonergic modulation can decouple aggression control from the broad CNS suppression typical of current antipsychotic regimens. These findings extend prior work showing that 5-HT_2B_ receptor upregulation is a feature of AD pathology and that its inhibition restores synaptic and circuit-level function disrupted by amyloid and tau oligomers.

Aggressive behavior emerges from the coordinated activity of multiple neurotransmitter systems, neuromodulators, intracellular signaling pathways, and hormones acting within distributed neural circuits that include the amygdala, hypothalamus, prefrontal cortex, and periaqueductal gray. Among these systems, serotonin has been consistently implicated as a key modulator of aggression, particularly in aggression control. Reduced serotonergic tone in cortical and limbic regions is strongly associated with increased impulsivity and escalated aggression [22]. The molecular bases of the initiation of aggressive attacks remain to be explored. In this framework, serotoninergic modulation might not be relevant to the latency to the first attack. The dissociation observed in our study—where MW073 reduced both the number and duration of attacks without affecting attack latency—supports the notion that the initiation of aggression and its subsequent escalation or maintenance rely on partially distinct neurobiological mechanisms. Specifically, inhibition of 5-HT2bR appears to preferentially modulate the expression and intensity of aggressive behavior once it has been initiated. This interpretation is consistent with a role for serotonergic signaling, and 5-HT_2B_Rs in particular, in regulating aggression severity, persistence, and impulse control, while leaving the initial triggering of aggression largely intact. Together, these findings suggest that MW073 attenuates the escalation of aggressive behavior without interfering with the fundamental motivational or perceptual processes that govern attack initiation. This could be of potential clinical significance in future planned clinical development, as it suggests that the first signs of episodic aggressivity would be justification for initiation of daily oral administration.

In this study, we did not detect aggressive attacks in female mice. Aggression-related behaviors in females may be less pronounced, delayed, or expressed in alternative social domains, and may also be influenced by hormonal status and experimental conditions. Thus, while aggressive attacks do not appear to be a feature in female *Tg2576* mice under our testing conditions, more subtle or context-dependent alterations in social behavior cannot be excluded. Given the absence of a detectable aggressive phenotype in female *Tg2576* mice under our experimental conditions, and to ensure sensitivity and robustness in the assessment of aggression-related outcomes, we therefore focused subsequent behavioral analyses on male animals, in which such behaviors are reliably observed.

Importantly, the present data reveal that 5-HT_2B_ signaling contributes not only to cognitive disturbances but also to aggression-like behaviors that model the agitation and irritability common in AD patients. This convergence across behavioral domains underscores the broader relevance of the 5-HT_2B_ pathway as a nodal regulator of stress-vulnerable neural circuits. As such, MW073 emerges as a precision-medicine candidate with a distinct safety and mechanistic profile from existing therapies, and *Tg2576* males provide a reliable platform for screening interventions targeting AD-associated agitation.

## 5. Conclusions

This study demonstrates that intervention MW073 reduces emerging AD-NPS aggressive behavior without inducing sedation or motor impairment. Taken in the context of our previous reports [12,16] and an accumulating body of knowledge about 5-HT_2B_R biology, 5-HT_2B_R-mediated signaling is implicated in the homeostasis and pathophysiology of a variety of chronic and acute neurological and neuropsychiatric disorders.

## 6. Patents

Saktimayee M. Roy, Erica Acquarone, Ottavio Arancio, and D. Martin Watterson are authors on patent applications filed by Northwestern and Columbia Universities that cover potential commercial use but not research use of MW073. The other authors have no relevant disclosures.

## Data Availability

The original contributions presented in this study are included in the article. Further inquiries can be directed to the corresponding authors.

## Abbreviations

The following abbreviations are used in this manuscript:

AD	Alzheimer’s disease
ADRD	AD-related disease
NPS	Neuropsychiatric syndromes
5-HT	5-hydroxytryptamine
5-HT_2B_R	5-HT_2B_ receptor
ALS	Amyotrophic lateral sclerosis
SNRIs	Norepinephrine reuptake inhibitors
PCF	Prefrontal cortex
